# Dual antiplatelet therapy and drug eluting stents: a marriage of convenience

**DOI:** 10.1186/1477-9560-5-15

**Published:** 2007-10-01

**Authors:** Juan Benezet-Mazuecos, Borja Ibanez, Juan J Badimon

**Affiliations:** 1 Cardiovascular Biology Research Laboratory, Cardiovascular Institute, Mount Sinai School of Medicine, New York, NY, USA

In 1977, a major breakthrough in cardiovascular medicine was introduced by Dr. Andreas Gruentzig. In September of that year, Gruentzig performed the very first percutaneous transluminal coronary angioplasty (PTCA) procedure in a human. Twenty-three years later, the coronary artery was still patent. [1] Despite the initial enthusiasm, some complications were clearly associated with the procedure, being restenosis and thrombosis the major problems to deal with. In the early nineties, bare metal stents (BMS) were introduced to avoid the so called "elastic recoil of the artery" leading to restenosis. Despite some improvements in the rates of restenosis and thrombosis, they were still very significant. The introduction of dual antiplatelet therapy (thyenopiridine on top of aspirin) substantially decreased the rate of stent thrombosis, being currently a rare complication occurring in less than 1% of the procedures. [2]

Despite the "elastic recoil" of the artery was abrogated with the stent deployment, there was still a non-depreciable rate of in-stent restenosis due to the neointimal proliferation inside the stents. In an attempt to fight the exaggerated proliferation after coronary stent implantation, our lab first used rapamycin in an animal model of restenosis showing the effectiveness of this antiproliferative drug in preventing this neointimal proliferation. [3] Soon after, stents locally delivering antiproliferative drugs (the so called drug eluting stents [DES]) were designed and tested in humans with impressive results in terms of in-stent restenosis inhibition. Two drug-eluting stents (DES), the Cypher stent (Cordis/Johnson & Johnson) and Taxus stent (Boston Scientific), were introduced. The Cypher and Taxus stents are produced by coating a stainless-steel stent with a thin layer of a non-erodable synthetic polymer containing either sirolimus or paclitaxel respectively. DES implantation resulted in a rate of restenosis below 10% (compared to 30% observed after bare metal stents implantation) [4-6], with a similar rate of in-stent thrombosis. [7,8] It is important to remind that dual antiplatelet therapy was mandatory for at least 3–6 months after DES implantation. However some initial warns were raised by few groups suggesting that DES do not undergo a complete re-endothelization. [9,10] It was just a matter of time to face, with striking surprise, the unexpected high rate of late (>30 days after stent deployment) and especially very late in-stent thrombosis (beyond 12 months from stent implantation) in DES compared to bare metal stents. [11,12] Further cause for concern came from the Basel Stent Cost-effectiveness Trial-Late Thrombotic Events (BASKET-LATE) data, which showed that among 746 DES or BMS patients who had dual antiplatelet therapy discontinued after the first six months, the rate of cardiac death or non-fatal MI over the following year was higher in patients with DES than BMS (4.9% versus 1.3%; p = 0.01), and that this was likely to be related to late stent thrombosis, which presented as death or MI in 88% of cases. [13] After this first report, a heated discussion began over the safety of DES, especially when the protective umbrella of dual antiplatelet therapy is discontinued. Dr. Camenzind presented in the WCC-2006 in Spain a meta-analysis of first generation DES in comparison to BMS accounting for a total of n = 878 sirolimus eluting stent (SES) vs n = 870 BMS (RAVEL, SIRIUS, C-SIRIUS and E-SIRIUS) and n = 1685 paclitaxel-eluting stents (PES) vs n = 1675 BMS (TAXUS II, IV-VI). The incidence – up to the latest available follow-up – of total mortality and Q-wave MI combined were 38% (SES) and 16% (PES) higher in DES as compared to control BMS (p-value: SES vs BMS: 0.03 ; PES vs BMS. 0.68). [14] Overall, it seems that experience has shown that the success of DES comes at a price: patients using DES could be trading restenosis, which is seldom life-threatening, for in-stent thrombosis, which may lead to death and myocardial infarction. Moreover, a number of reports imply that thrombosis rates of DES may even be higher in the "real world" than in clinical trials where stents are used "on-label". Today it is considered that as much as 60% of coronary percutaneous procedures are "off-label". When DES are used "off-label", it is estimated that the rates of thrombosis are higher. On the other hand, although data from large registries and meta-analyses of randomized trials indicate a higher risk for DES thrombosis, others recent studies claim the absence of such increased risk. [15] The controversy is open.

Three are the major players involved in the pathological processes leading to late in-stent thrombosis: the stent coated with an antiproliferative drug, the vulnerability (thrombogenicity) of the patient's blood, and the antiplatelet therapy.

Several factors related with the stents are associated with an increased risk of thrombosis, including the procedure itself (stent malapposition and/or underexpansion, number of implanted stents, length, persistent slow coronary blood flow, and dissections) and stent design (materials, strut thickness and polymer type). The coating of stents with antiproliferative (sirolimus) and/or cytostatic (paclitaxel) drugs should have the additional advantage of inhibiting the proliferation of vascular cells. [10]

Reendothelialization occurs after vascular injury and similarly after stent placement. But then two questions arise since damaged and/or dysfunctional endothelium is directly related with the development and progression of atherosclerosis [16,17]: Could the DES selectively inhibit the proliferation and migration of smooth muscle cells without affecting the growth of the endothelial cells? And could it be possible for DES to exert their antirestenotic effects without affecting the neo-endothelialization of the struts? In vitro, rapamycin and paclitaxel not only inhibit proliferation and migration of vascular smooth muscle cells but equally suppress endothelial cells, thereby potentially impairing reendothelialization. A post-mortem study comparing coronary segments from patients after DES and BMS implantation revealed delayed arterial healing and poorer endothelialization after DES compared with BMS implantation of similar duration. [10] Thus, current evidence suggests delayed reendothelialization and arterial healing after implantation of DES compared with BMS, resulting in potentially enhanced thrombogenicity. Many factors influence the healing process and vary with individual risk factors. Coating of stents with substances that potentially facilitate reendothelialization may represent a novel therapeutic approach. Significant advances in material engineering are poised to produce biodegradable stents. [18]

Several risk factors correlate with "vulnerable/hyperreactive blood" (diabetes, smoking, hyperlipidemia, inflammatory states, cathecholamines...). Also, several patient-related factors have been associated with the development of in-stent thrombosis, including low ejection fraction, diabetes mellitus, advanced age, renal failure, and acute coronary syndromes. In particular, in this last situation it could be due to delayed healing, lack of endothelialization, and presence of a pronounced inflammatory and thrombogenic environment combined by enhanced platelet reactivity. Furthermore, certain lesion characteristics (bifurcation complex or in-stent restenosis lesions) are reported to be associated with an increased risk of stent thrombosis. [11,19]

The majority of the retrospective studies on the rate of late in-stent thrombosis have identified the window of 6 to 9 months post stenting as the peak of more incidence. The majority of the studies using multivariate analysis have identified the discontinuation of dual antithrombotic therapy as the principal parameter responsible for stent thrombosis. [20,21] Stents are foreign bodies in the vessel wall and thus induce platelet adhesion and activation of the coagulation cascade. Furthermore, high-pressure implantation induces significant vascular injury, with exposure of thrombogenic molecules of the subintima and media (including plaque material) to the blood stream. As a consequence, only potent platelet inhibition made the procedure feasible. [22] The association between late thrombosis with delayed stent coverage and cessation of dual antithrombotic therapy strongly suggest the need for a longer period of combination therapy. On the other hand, longer administration of dual therapy is hampered by its higher cost, patient compliance and the possibility of increased bleeding complications. The appropriate duration of the long-term antiplatelet regimen for prevention of DES thrombosis remains to be assessed in randomized prospective trials; at present, a course of 12 months of dual-antiplatelet therapy may be considered. [23]

Therefore, in an example of individualized medicine, many factors should be taken into account for the selection of the type of stent to be implanted. Among these factors, the patient characteristics (diabetes, left ventricular ejection fraction ...), the severity of the disease and the characteristics of the lesions, and finally the possibility and/or compliance to a prolonged antithrombotic treatment. What seem to be clear is that a longer period of dual anti-platelet therapy is needed for patients undergoing DES implantation than for those receiving a bare metal stent. However, at this point it is completely unknown how longer that period should be. Despite the current recommendations from the AHA/ACC suggest 12 months of dual antiplatelet therapy, some experts highly suggest a chronic dual antiplatelet therapy until new data with longer follow up periods is available.
